# Effect of a single high dose vitamin A supplementation on the hemoglobin status of children aged 6–59 months: propensity score matched retrospective cohort study based on the data of Ethiopian Demographic and Health Survey 2011

**DOI:** 10.1186/1471-2431-14-79

**Published:** 2014-03-21

**Authors:** Samson Gebremedhin

**Affiliations:** 1 School of Public and Environmental Health, Hawassa University, Hawassa, Ethiopia

**Keywords:** Vitamin A supplementation, Anemia, Hemoglobin

## Abstract

**Background:**

Vitamin A deficiency can cause anemia as the nutrient is essential for hematopoiesis, mobilization of iron store and immunity. Nevertheless, clinical trials endeavored to evaluate the effect of Vitamin A Supplementation (VAS) on hemoglobin concluded inconsistently. Accordingly, the objective of the current study is to assess the effect of single high dose VAS on the hemoglobin status of children aged 6–59 months.

**Methods:**

The study was conducted based on the data of Ethiopian Demographic Health Survey 2011. The data from 2397 children aged 6–59 months who received a single dose of 30 or 60 mg of VAS (depending on age) in the preceding 6 months were matched with similar number children who did not receive the supplement in the reference period. The matching was based on propensity scores generated from potential confounders. Distributions of hemoglobin concentration and risks of anemia were compared between the groups using paired t-test, matched Relative Risk (RR) and standardized mean difference.

**Result:**

The supplemented and non-supplemented groups were homogeneous in pertinent socio-demographic variables. Compared to propensity score matched non-supplemented children, those who received vitamin A had a 1.50 (95% CI: 0.30-2.70) g/l higher hemoglobin concentration (*P = 0.014*). In the supplemented and non-supplemented groups, the prevalences of anemia were 46.4% and 53.9%, respectively. VAS was associated with a 9% reduction in the risk of anemia (RR = 0.91 (95% CI: 0.86-0.96)). Stratified analysis based on household wealth status indicated that the association between VAS and hemoglobin status was restricted to children from the poor households (RR = 0.74 (95% CI: 0.61-0.90)). Effect size estimates among all children (Cohen’s d = 0.07) and children from poor households (d = 2.0) were modest.

**Conclusion:**

Single high dose VAS among Ethiopian children 6–59 months of age was associated with a modest increase in hemoglobin and decrease in risk of anemia. Household wealth status may modify the apparent association between VAS and hemoglobin status.

## Background

Anemia is a global public health problem affecting both developing and developed countries. It poses serious consequences for human health including increased risk of maternal and child mortality [1]. According to World Health Organization (WHO), anemia affects 24.8% of the world population and the burden is substantially high among preschool-aged children (47.4%), pregnant women (41.8%) and women of reproductive age (30.2%) [1]. In 2002 Iron Deficiency Anemia (IDA) was identified as one of the major contributing factors to the global burden of disease [2].

Over years several studies documented the public health significance of anemia in Ethiopia. The recent Ethiopia Demographic and Health Survey (EDHS) 2011 reported 44.2%, 22.0% and 16.6% prevalence of anemia among preschool-aged children, pregnant women and non-pregnant women, respectively [3]. The previous EDHS 2005 also reported relatively higher (53.5%, 30.6% and 26.6%) prevalences in the aforementioned three population groups, consecutively [4].

Several factors, both nutritional and non-nutritional, are known to contribute to the onset of anemia. However, nutritional anemia is the most widespread type. Especially IDA is estimated to contribute to approximately 50% of the global burden of anemia – though the proportion may vary according to local situations. Other micronutrient deficiencies including folate, vitamin B-12, vitamin C, Vitamin A (VA), zinc and cooper are also linked with anemia [1,5].

The relationship between Vitamin A Deficiency (VAD) and anemia has been known for many decades now [6]. So far various pathophysiological mechanisms had been postulated. Vitamin A appears to enhance hematopoiesis and mobilization of iron store possibly through increasing circulating erythropoietin [6,7]. VA could also prevent anemia associated with infection via its immune-modulatory effect [6]. Vitamin A deficiency might also alter absorption and storage of iron [5].

Several observational studies witnessed significant association between hemoglobin and various VA status indicators [6]. Reasonable number of Randomized Controlled Trials (RCTs) based on daily or weekly VA Supplementation (VAS) have also concluded likewise [8-12]. However, RCTs based on single high dose VAS concluded equivocally. Studies in Thailand [13], Indonesia [14] and Morocco [7] reported positive effects; whereas, those in Peru [15] and Thailand [16] found no association.

In settings where VAD is a public health problem, the WHO recommends for routine and high dose VAS every 4–6 months for children 6–59 months [17]. This is based on the knowledge that a single, large dose of VA is well absorbed in the liver and can be mobilized over an extended period of time as required. The recently revised WHO guideline emphasizes on the significance of VAS for the reduction of childhood mortality, xerophthalmia and nutritional blindness [17]. The systematic review by Cochrane collaboration also concluded that VAS reduces all-cause childhood mortality by 24% [18].

Accordingly the current study, based on the data of EDHS 2011, was carried out in order to evaluate the effect of routine high dose VAS on hemoglobin status of children aged 6–59 months. The aforementioned dataset was selected, considering the fact that the prevalences of VAD and anemia are known to be high in Ethiopia [3,4,19] and the country is also implementing large scale semi-annual VAS for children aged 6–59 months.

## Methods

### Study design

The current study – a retrospective cohort by design – is a secondary data analysis of the Ethiopia Demographic and health survey (EDHS) carried out in 2011. Children aged 6–59 months who received and did not receive VAS in the preceding 6 months of the survey were identified and matched using propensity score matching technique. Ultimately mean hemoglobin concentration and anemia status determined at the time of the survey were compared between the two study groups.

### Study setting

Ethiopia is among the least developing countries in the world with Gross Domestic Product (GDP) per capita of 1,200 USD [20]. Of approximately 80 million Ethiopians, 84% live in rural areas where access to social services is limited [21]. The country’s economy is dependent on agriculture and 29.2% of the population lives below the poverty-line [20]. Despite the recent improvements in health indicators, infant and under five mortality rates (50 and 88 deaths per 1,000 live births, respectively) remain high and the life expectancy at birth does not exceed 57 years [3,20]. Malnutrition remains a major problem as 44%, 29% and 10% of the preschool-aged children are stunted, underweight and wasted, respectively [3]. Widespread poverty, food insecurity and limited access to social services have contributed to the high burden of ill-health in the country [20].

Parallel to the recommendation of WHO, Ethiopia implements routine VAS for children 6–59 months. According to the national guideline, children aged 6–11 and 12–59 months are given 100,000 and 200,000 international units of VA (i.e. 30 and 60 mg of retinol), respectively, on semi-annual basis [22]. Usually VA capsules are distributed through Enhanced Outreach Strategy/Community Health Days (EOS/CHD) campaigns. Other services provided during the campaign include deworming of children 24–59 months and nutritional screening of children 6–59 months. VAS is also conducted during routine vaccination and sick child visit of health institutions. According to DHS 2011 the coverage of VAS in the aforementioned age group in the country was 53.1%.

### Sampling design

The EDHS 2011 applied two stage cluster sampling technique. Enumeration Area (EA) — a cluster that conventionally encompasses 150–200 adjacent households — was the first stage sampling unit. The original survey included 624 EAs, 187 in urban and 437 in rural areas. Ahead of the survey, a complete listing of households was carried out in each of the EAs and eventually 17,817 households were randomly selected [3].

**Figure 1:**
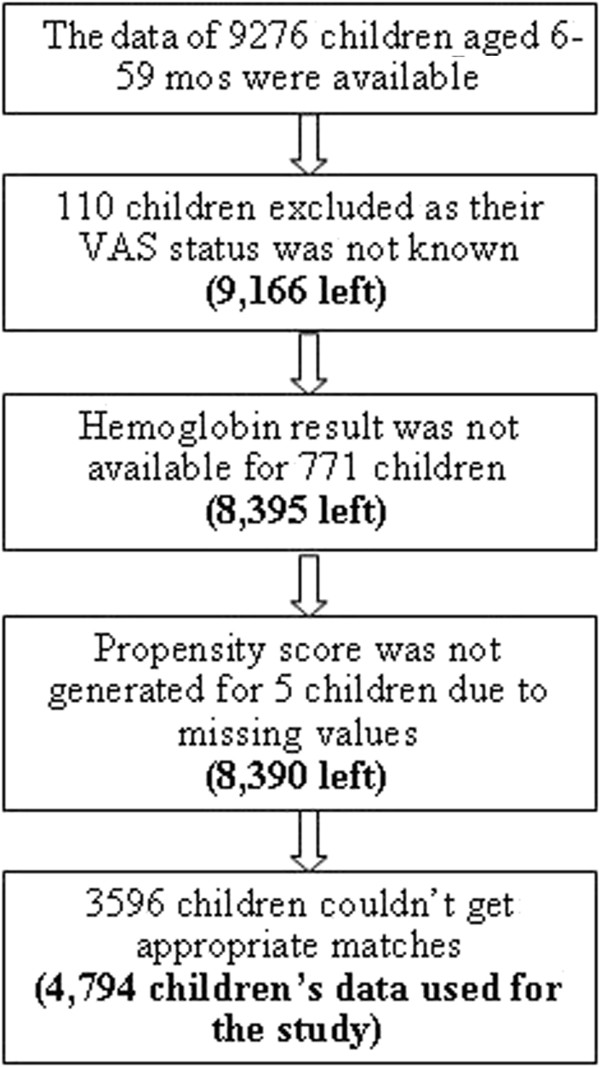
Flowchart of the study.

For the current analysis, the data of 9,276 children aged 6–59 were available. However, for various reasons the data of only 4,794 children were used for the analysis. Reasons for exclusion were; lack of information about the VAS status or hemoglobin concentration of the children, missing values for the variables needed to generate propensity score and unable to find appropriate matches (Figure 1). Children included and excluded from the study were not significantly different in terms of basic socio-demographic variables include age, sex, place of residence (urban/rural), wealth index and parents’ educational status (P > 0.05).

### Power calculation

Power to detect a difference in the prevalence of anemia was computed based on the available number supplemented and non-supplemented children in the dataset and the prevalences of anemia found in the two groups. The computation was made using the online calculator called StatsToDo which is designed for matched study design [23]. The inputs of the calculation were: 95% confidence level; 2,397 pairs of supplemented and non-supplemented subjects; 46.7% and 53.9% prevalences of anemia in supplemented and non-supplemented children; and one-to-one ratio between the two study groups. Eventually the power was computed as 79.8% and it was judged to be optimal.

### Data collection

The EDHS 2011 data were collected from December 2010 to June 2011 using trained and experienced data collectors. The survey used standard MEASURE DHS questionnaire adapted to the Ethiopian context. The questionnaire was finalized in English and translated to three major local languages. Prior to the fieldwork, the tools were pretested and all necessary modifications were made [3].

### Exposure and outcome ascertainments

During the survey VAS status of the children was determined by showing their mothers/primary caregivers a VA capsule and enquiring whether their children had been given a similar one in the preceding 6 months [3]. Hemoglobin concentration was determined via portable HemoCue analyzer using a drop of capillary blood and the concentration was adjusted for altitude according to the recommendation of Centers for Disease Prevention and Control (CDC) [24]. The cutoff points applied to define anemia were: mild (100–109 g/l), moderate (70–99 g/l) and severe (< 70 g/l).

### Matching of VA supplemented and non-supplemented children

The propensity score is the conditional probability of assignment to a particular treatment given a vector of observed covariates [25]. Propensity score matching refers to the pairing of treatment and control units with similar values on the propensity score. It is an important tool for causal inference in retrospective cohort and quasi-experimental studies in which random assignment of treatments is impossible and asymmetry of treatment groups is likely. Propensity score matching avoids selection bias associated with covariates used to predict the score [26].

In the current analysis propensity scores were generated via binary logistic regression model that compute the probability of receiving high dose VA, as a function of eleven factors/covariates. The factors/covariates were wealth index, parents’ educational status, place of residence (urban or rural), age of the child, sex of the child, number of preschool age children in the household, household’s usual source of drinking water (improved or unimproved), household’s excreta disposal method (improved or unimproved), vaccination status of the child, and deworming treatment of the child within 6 months of the survey. Child illness related variables were not considered in generating the propensity scores as they are potential mediator factors between VAS and hemoglobin status.

Eventually, every VA supplemented child was matched with a non-supplemented one using a variant of propensity score matching method called Caliper matching (i.e. matching to a control with the nearest propensity score that is within a predefined width). The caliper width was set as 0.2 of the Standard Deviation (SD) of the logit of the propensity score [27]. Ultimately 2,397 VA supplemented and 2,397 non-supplemented children were matched.

### Data management and analysis

The dataset was downloaded from Measure DHS website and cleaned using SPSS 20.0 software. The data were subsequently exported to Stata SE 11 for analysis. Mean hemoglobin concentrations in supplemented and non-supplemented children were compared using paired t-test. The association between VAS status and anemia was determined via McNemar’s Chi-square and matched Relative Risk (RR). Both were generated using the Stata MCC command modified for matched cohort design [28]. Statistical significance was set at *P* value of 0.05. Effect Size (ES) calculation was made using the standardized mean difference method. Prior to analysis the assumptions of McNemar’s Chi-square and t-tests had been checked.

In order to assess the effectiveness of the propensity score matching, the comparability of the two treatment groups on the variables used to generate the propensity score was checked using paired t- or McNemar’s Chi-square- tests. Further, the similarity of the groups based on other selected variables including dietary diversity score, meal frequency and breastfeeding was assessed. Dietary diversity score was calculated according to the recommendation of the WHO [29].

Wealth index was computed as a composite indicator of living standard based on 18 variables related to ownership of selected household assets, size of agricultural land, quantity of livestock and materials used for housing construction. The computation was made using principal component analysis. Initially the analysis generated six principal components and a single continuous variable was generated by summing up the principal components into one. Tertiles of wealth index (poor, middle and rich) were generated using the composite score.

### Ethical consideration

The dataset was accessed after securing permission from Measure DHS organization. During the survey, the data were collected in confirmation of national and international ethical guidelines. Ethical clearance for the survey was provided by the Ethiopian Health and Nutrition Research Institute (EHNRI) review board, the National Research Ethics Review Committee (NRERC) at the Ministry of Science and Technology, the Institutional Review Board of ICF International, and the CDC [3].

## Results

### Background characteristics of the study subjects

A total of 2,397 pairs of VA supplemented and non-supplemented children were included in the analysis. In order to evaluate the overall effectiveness of the propensity score matching, the basic characteristics of the two groups were compared using paired t- or McNemar’s Chi-square- tests. The mean (±SD) propensity score was virtually identical for the two groups (0.50 ± 0.17 for both).

The mean (±SD) age of the children in supplemented and non-supplemented groups were 31.6 (±15.3) and 31.7 (±15.9) months (*P = 0.718*). The boys to girls ratios were 1.03 and 1.02, consecutively, (*P = 0.974*). Likewise, the study groups were comparable with respect to socio-economic status indicators including parents’ educational status, place of residence, household wealth index and household size (*P > 0.05*). Access to improved water source and sanitary facility, proportion of children who completed vaccination, and proportion of children who received deworming tablets in the preceding 6 months of the survey, were also similar. Further, among children aged 6–23 months, proportion who were breastfeeding during the survey and mean food frequency and dietary diversity score in the preceding day of the study were comparable (*P > 0.05*) (Table 1).

**Table 1:** Comparison of the characteristics of vitamin A supplemented and non-supplemented children aged 6–59 months, Ethiopia, 2010

**Variables**	**VAS status**		**Test statistic and P values for paired t or McNemar’s test**
	**Supplemented (n = 2,397)**	**Non-supplemented (n = 2,397)**	
Mean child age in months (mean (±SD))	31.6 (±15.3)	31.7 (±15.9)	*t* = 0.36, *P* = 0.718
Proportion of female children (%)	49.4	49.4	*χ2* = 0.00, *P* = 0.974
Proportion of mothers who had any formal education (%)	30.1	28.0	*χ2* = 3.79, *P* = 0.056
Proportion of fathers who had any formal education (%)	47.0	46.6	*χ2* = 0.08, *P* = 0.799
Proportion of urban residents (%)	14.0	14.5	*χ2* = 0.29, *P* = 0.629
Mean wealth index score (mean (±SD))	-0.36 (±0.07)	-0.36 (±0.07)	*t* = 0.29, *P* = 0.770
Proportion of households with improved water source (%)	51.1	51.4	*χ2* = 1.11, *P* = 0.317
Proportion of households with improved sanitary facility (%)	11.1	10.9	*χ2* = 0.09, *P* = 0.806
Proportion of children who received deworming tablet within 6 months (%)	9.6	9.3	*χ2* = 3.27, *P* = 0.119
Household size (mean (±SD))	6.18 (±2.32)	6.18 (±2.33)	*t* = 0.06, *P* = 0.995
Proportion of children 12–59 months who completed vaccination (%)^♦^	64.2	64.5	*χ2* = 1.26, *P* = 0.337
Proportion of children 6–23 months who were breastfeeding during the survey^*^	91.7	89.7	*χ2* = 0.47, *P* = 0.492
Dietary diversity score among children 6–23 months (mean (±SD))^*^	1.29 (±1.07)	1.21 (±1.07)	*t* = 0.84, *P* = 0.401
Mean feeding frequency among children 6–23 months (mean (±SD))^*^	1.78 (±1.67)	1.73 (±1.60)	*t* = 0.43, *P* = 0.662

♦n = 1,573 pairs of children.

^*^n = 898 pairs of children.

### Vitamin A supplementation and anemia

The mean (±SD) hemoglobin levels in supplemented and non-supplemented children were 107.5 (±17.9) and 106.0 (±23.8) g/l, respectively, reflecting a significant mean difference of 1.50 (95% CI: 0.30-2.70) g/l in favor of the supplemented group (*t = 2.471*, *P = 0.014*) (Table 2).

**Table 2:** Mean hemoglobin concentration in vitamin A supplemented and non-supplemented children aged 6–59 months, Ethiopia 2010

**Mean hemoglobin concentration (g/l)**	**Mean (**±**SD)**
All children (n = 4794)	106.7 (±21.1)
VA supplemented children (n = 2397)	107.5 (±17.9)
VA non-supplemented children (n = 2397)	106.0 (±23.8)
Paired mean difference (supplemented - non supplemented) (n = 2397)	1.5 (±21.1)

Amongst supplemented children, the prevalence of anemia was 46.4% (95% CI: 44.4-48.4%). About 20.3%, 22.1% and 3.2% had mild, moderate and severe anemia, respectively. Alternatively, among non-supplemented children, the prevalence of any form of anemia was 53.9% (95% CI: 51.9-55.9%) and 3.9%, 27.8% and 3.9% had mild, moderate and severe anemia, respectively. In the VA supplemented group, the risk of anemia was significantly reduced, represented by a RR of 0.91 (95% CI: 0.86-0.96) (Table 3).

**Table 3:** Pattern of anemia among 2397 paired vitamin A supplemented and non-supplemented children aged 6–59 months, Ethiopia, 2010

		**Supplemented**		**Total**
		**Normal**	**Anemic**	
**Non-supplemented**	**Normal**	558	541	1099
	**Anemic**	653	645	1298
**Total**		1211	1186	2397

Matched RR = 0.91 (95% CI: 0.86-0.96).

McNemar’s *χ2* = 10.51, *P* = 0.001.

### Effect modification by household wealth status

The association between VAS and anemia was independently computed across the three wealth strata (poor, middle and rich). The analysis indicated that the significant association was only restricted in the ‘poor’ household wealth stratum (RR = 0.74 (95% CI: 0.61-0.90)). In contrast, the association was marginal in the middle (*P = 0.059*) and insignificant in the rich wealth strata (*P = 0.630*) (Table 4).

**Table 4:** The association between VAS and anemia among children aged 6–59 months across three household wealth strata, Ethiopia, 2010

**Wealth tertiles**	**Number of matched children**^♦^	**RR (95% CI) in VA supplemented group**	**McNemar’s χ**^**2 **^**test**
Poor	331	0.74 (0.61-0.90)*	χ^2^ = 9.48, P = 0.002*
Middle	311	0.86 (0.74-1.00)	χ^2^ = 3.55, P = 0.059
Rich	329	0.96 (0.82-1.12)	χ^2^ = 0.32, P = 0.630

^♦^Number of matched children both selected from the respective wealth category.

*Statistically significant.

Likewise the mean hemoglobin differences between matched supplemented and non-supplemented children in the poor, middle and rich wealth categories were 5.4 (±26.8), 3.1 (±25.8) and 0.3 (±23.7) g/l, respectively. Pared t-test analysis was significant only in the poor tertile (*P* = 0.000). Comparison of the three mean differences using one way ANOVA showed statistically significant global difference (*P = 0.039*) and Tukey’s post-hoc test detected significant difference between poor and rich tertiles (Table 5).

**Table 5:** Mean hemoglobin difference between matched vitamin A supplemented and non-supplemented children aged 6–59 months across three household wealth strata, Ethiopia, 2010

**Wealth tertiles**	**Mean (**±**SD) hemoglobin paired difference**^**• **^**(g/l)**	**Paired t statistic and *p *** **value**	One Way ANOVA^**^
Poor	5.4 (±26.8)	*t* = 3.64, *P* = 0.000^*^	*F* = 3.24, *P* = 0.039^*^
Middle	3.1 (±25.8)	*t* = 1.66, *P* = 0.979	
Rich	0.3 (±23.7)	*t* = 0.26, *P* = 0.796	

^**•**^Supplemented minus non-supplemented.

*Statistically significant.

**Used as a measure of heterogeneity of effects.

### Evaluation of the practical significance of VAS in the prevention of anemia

In the evaluation of the effect of an intervention on an outcome, along with statistical level of significance, it’s important to appraise its practical significance using effect size estimates. This is particularly important in studies involving large sample sizes as they are likely to detect statistically significant difference even in the presence of trivial treatment effect.

In the current study, the effect sizes computed based on standardized mean differences (Cohen’s d) among all children and children from poor households were of 0.07 and 0.20, respectively. As compared to the cutoff points recommended by J Cohen [30], the effect size estimates were modest.

## Discussion

In the current study a relatively small but statistically significant hemoglobin increase of 1.5 g/l was observed in VA supplemented group. The increment is minimal as compared to results from three previous RCTs that had used daily or weekly VAS. The RCTs conducted in Tanzania (1.5 mg VA for 3 days a week for 3 months) [8], Belize (1.0 mg per week for 6 months) [9] and Guatemala (3.0 mg VA daily for 2 months) [10] reported statistically significant 9.9, 8.0 and 6.1 and g/l hemoglobin increments in VA supplemented children, respectively. Compared to the effects reported from these RCTs, the small treatment effect estimated from the current study might be due to variation in type of VAS regimen (i.e. daily, weekly or semi-annual supplementation). Though no study so far compared the effectiveness of various VAS regimens, few studies on other micronutrients documented better physiological responses in more frequent supplementation regimens [31-33].

Clinical trials based on high dose VA supplementation in children have generated mixed findings with respect to the impact on hemoglobin. In Peru [15] and Thailand [16] 30 mg and 60 mg VAS respectively, did not yield significant hemoglobin improvements. Another study in Thailand [13] witnessed a significant but relatively slim 3 g/l increment following administration of single 60 mg VA supplement. In Indonesia, 60 mg VAS did not show significant effect among clinically normal children but significantly increased the hemoglobin concentration by 7 g/l among anemic children [14]. In Morocco, two 60 mg VA supplementations given 5 months apart increased hemoglobin by 6 g/l [7]. The findings of the current study along with the aforementioned trials may indicate that high dose VAS has less remarkable effect on blood hemoglobin level than daily or weekly regimens.

In the current study, the relatively weak association observed between VAS and hemoglobin/anemia can also be due to sub-optimal dietary iron intake of the study subjects. As VA is assumed to increase hemoglobin level principally through facilitating hematopoisis and mobilization of iron store [5,6], VAS in the absence of optimal iron status might not illustrate pronounced effect on hemoglobin concentration. According to EDHS 2011, among children aged 6–23 months only 13.3% consumed iron rich foods in the preceding day of the survey and among children 6–59 months only 6.0% had any form of iron supplementation in the previous one week of the survey [3].

The stratified analysis based on household wealth status indicated that the significant association between VAS and anemia was only restricted to children from the poor households. The strength of association between the two variables uniformly reduced across the three wealth strata — poor (RR = 0.74), middle (RR = 0.86) and rich (RR = 0.96). This might be due to the reason that children from the poor families would have less access to VA rich foods hence they tend to benefit more from the supplementation. Conversely among children from households of higher socio-economic means, the protective effect of VAS would be minimal as they may already been adequate in the baseline VA status. So far no trial examined the modifying effects of household economic status on responses to micronutrient supplementation among children. But a study among pregnant women in China reported that in women from the poorest tertile of the socio-economic status, micronutrient supplementation significantly reduced risk of low birthweight and early neonatal mortality rate; however, similar effects had not been seen among women from the wealthier households [34].

In Ethiopia, VAS is usually given to children along with other services like vaccination and mass-deworming. These services can also have independent positive effect on hemoglobin and could potentially confound the association between VAS and anemia. However, in the current study the confounding effect might not be a serious concern as both of the variables had been used to generate the propensity score for matching.

Some limitations need to be considered while interpreting the findings of the study. Primarily the ascertainment of the VAS status was entirely based on mothers’ recall. This makes the study liable to recall and misclassification bias and it can result in under- or over-estimation the actual strength of association. Though the study used propensity score matching to balance VA supplemented and non-supplemented groups based on selected covariates, still confounding can happen due to lack of comparability in other unmeasured characteristics. Further, presumably there is some delay between VAS and its effect on hemoglobin. However, in the current study the association was measured regardless of the time gap between the supplementation and hemoglobin determination, consequently this can result in under estimation of the association. The large number of subjects excluded from the study due to lack of appropriate matches can also be considered as a drawback of the propensity score matching method. In general, as the study is observational, the strength of the evidence might not be up to the level of RCTs.

## Conclusion

Single high dose VAS among Ethiopian children 6–59 months of age was associated with a modest increase in hemoglobin and decrease in risk of anemia. Household wealth status may modify the apparent association between VAS and hemoglobin status.

## Competing interests

The author declares that he has no competing interests.

## Authors’ contributions

SG exclusively conducted the data analysis and write-up of the manuscript.

## Authors’ information

SG is currently working as an assistant professor of public health at School of Public and Environmental Health, Hawassa University, Ethiopia.

## Pre-publication history

The pre-publication history for this paper can be accessed here:

http://www.biomedcentral.com/1471-2431/14/79/prepub
